# Identification of *flgZ* as a Flagellar Gene Encoding a PilZ Domain Protein That Regulates Swimming Motility and Biofilm Formation in *Pseudomonas*

**DOI:** 10.1371/journal.pone.0087608

**Published:** 2014-02-04

**Authors:** Francisco Martínez-Granero, Ana Navazo, Emma Barahona, Miguel Redondo-Nieto, Elena González de Heredia, Irene Baena, Irene Martín-Martín, Rafael Rivilla, Marta Martín

**Affiliations:** Departamento de Biología, Universidad Autónoma de Madrid, Madrid, Spain; Laurentian University, Canada

## Abstract

Diguanylate cyclase and phosphodiesterase enzymatic activities control c-di-GMP levels modulating planktonic versus sessile lifestyle behavior in bacteria. The PilZ domain is described as a sensor of c-di-GMP intracellular levels and the proteins containing a PilZ domain represent the best studied class of c-di-GMP receptors forming part of the c-di-GMP signaling cascade. In *P. fluorescens* F113 we have found two diguanylate cyclases (WspR, SadC) and one phosphodiesterase (BifA) implicated in regulation of swimming motility and biofilm formation. Here we identify a *flgZ* gene located in a flagellar operon encoding a protein that contains a PilZ domain. Moreover, we show that FlgZ subcellular localization depends on the c-di-GMP intracellular levels. The overexpression analysis of *flgZ* in *P. fluorescens* F113 and *P. putida* KT2440 backgrounds reveal a participation of FlgZ in Pseudomonas swimming motility regulation. Besides, the epistasis of *flgZ* over *wspR* and *bifA* clearly shows that c-di-GMP intracellular levels produced by the enzymatic activity of the diguanylate cyclase WspR and the phosphodiesterase BifA regulates biofilm formation through FlgZ.

## Introduction

The turn-over of the messenger molecule c-di-GMP modulates lifestyles in a diversity of bacteria. c-di-GMP levels have been shown to define the planktonic/sessile behavior of bacterial cells, reviewed in [1]. High levels of c-di-GMP down regulate motility and lead to biofilm formation, while low levels induce a planktonic lifestyle with highly motile cells. The turn-over of c-di-GMP is controlled by enzymes presenting diguanylate cyclase (DGC) activity and enzymes with c-di-GMP specific phosphodiesterase (PDE) activity. DGC activity is present in proteins containing a GGDEF domain while two types of PDEs have been found: proteins with an EAL domain or proteins with a HD-GYP domain. Genes encoding proteins with these domains are ubiquitous in bacterial genomes and are frequently present in large numbers in a single genome. Proteins containing both GGDEF and EAL domains are also frequent and in some cases, one of the domains could be non-catalytic, but participates in alosteric regulation or as a c-di-GMP sensor [2]. DGCs and PDEs are integrated in multiple regulatory pathways and other domains able to specifically sense c-di-GMP have been described (reviewed in [3]). One of these domains is the PilZ domain that is present in proteins implicated in different pathways such as cellulose production and pili formation [4].

c-di-GMP is implicated in the regulation of motility and biofilm formation in pseudomonads. Several proteins implicated in c-diGMP synthesis and degradation, have been described in *P. aeruginosa*, *P. fluorescens* and *P. putida*. In *P. aeruginosa* PA14 a system formed by a membrane bound DGC, SadC, and a PDE, BifA, has been shown to reciprocally modulate biofilm formation and swarming motility by affecting exopolysaccharide production and flagellar function [5], [6]. In this strain, a *sadC* deletion mutant presented altered flagellar reversal while swimming in a viscous medium [6].

In *P. putida*, proteins with GGDEF and EAL domains have been shown to modulate biofilm formation and dispersal [7]. Also in *P. putida*, the only response regulator which contains a GGDEF and an EAL domain, rup4959, is upregulated in the rhizosphere and influences biofilm formation, swimming and swarming motility and rhizosphere colonization [8].

A mechanism for c-diGMP activity in biofilm formation has been described in *P. fluorescens* Pf0-1 [9]: c-diGMP binding to the LapD protein leads to sequestering of the LapG protease, avoiding the cleavage of the adhesin LapA. A systematic analysis of DGCs has been performed in the same strain [10]. In this study four DGCs were shown to be implicated in biofilm formation, since mutations in these genes reduced the amount of biofilm formed. Interestingly one of these proteins affected the localization of the adhesin LapA, another affected swimming motility and a third affected both LapA and motility. c-diGMP is also implicated in the regulation of swimming and swarming motility and biofilm formation in *P. fluorescens* F113, a strain with biocontrol activity [11] and a model bacterium for rhizosphere colonization [12], [13], [14], [15]. We have previously shown that different pathways control swimming motility in this bacterium [16]. Flagella synthesis is regulated through the Gac/SadB pathway, being SadB a protein with a modified HDOD domain that might act as a sensor for c-di-GMP [16]. Furthermore WspR, a protein with a GGDEF domain and with DGC activity in *Pseudomonas aeruginosa* [17], several environmental isolates [18] and *P. fluorescens* SBW25 [19], regulates swimming motility through other pathway which is independent of flagella synthesis [16]. WspR also regulates swarming motility and biofilm formation in *P. fluorescens* F113 and its *wspR* mutant is defective in biofilm formation but possesses swarming motility under conditions than the wild-type strain does not [12].

The F113 genome encodes more than thirty proteins with GGDEF, EAL, HD-GYP and PilZ domains [20], [21]. In this study we have analyzed the role of WspR and the F113 SadC and BifA orthologs in order to investigate the role of c-di-GMP in the control of swimming motility and biofilm formation in *P. fluorescens* F113. We have also identified a gene encoding a protein containing a PilZ domain and located in a flagellar operon as a participant in the regulation of motility and biofilm formation in this bacterium.

## Results

### *flgZ* is a Flagellar Gene

**Figure 1:**
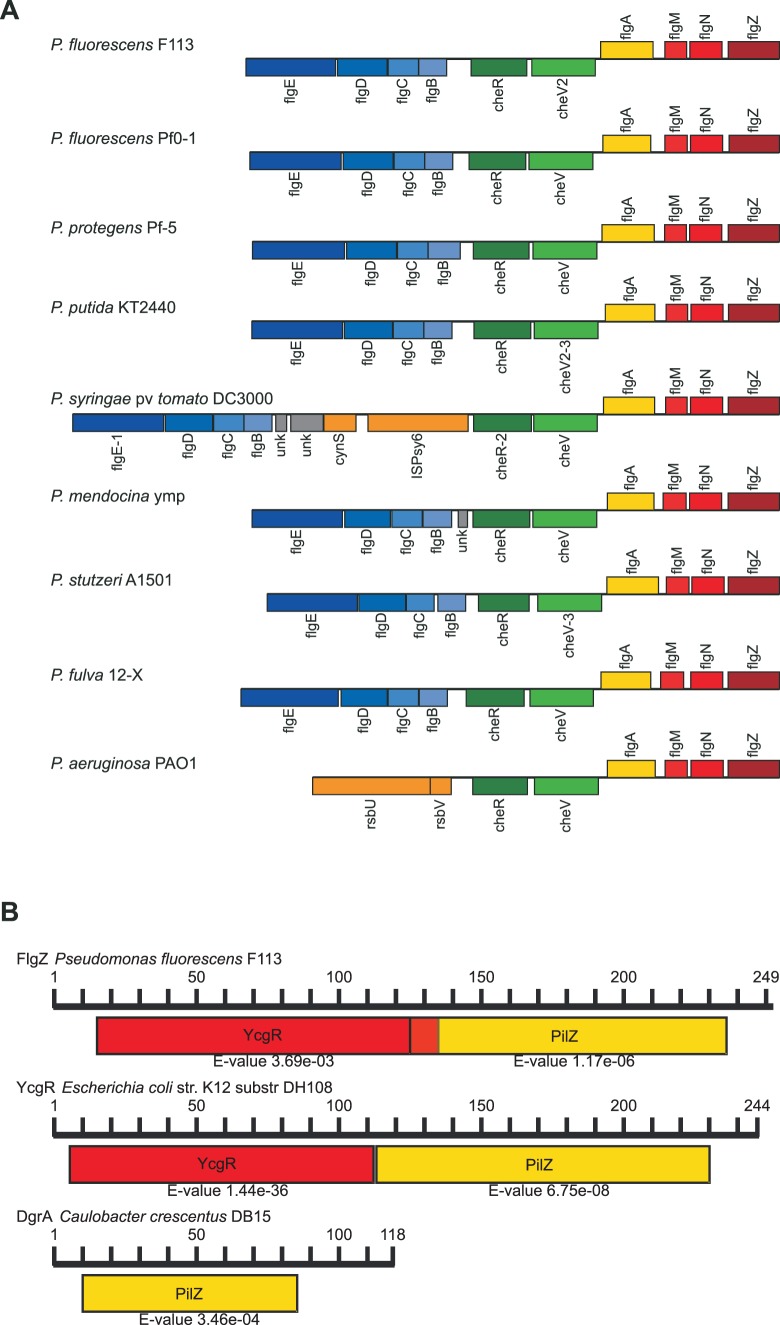
*flgZ* is a conserved gene that encodes a PilZ protein homologous to YcgR. A. Alignment of the genomic region which contains the *flgZ* gene in representative strains across the genus *Pseudomonas*. In all the strains the *flgZ* gene lies within a flagellar genes region, located downstream the *flgMN* genes. Other *flg* and *che* genes are also conserved in all the strains. B. Domain architecture of the FlgZ protein compared to flagellar brake proteins YcgR from *Escherichia coli* and DgrA from *Caulobacter crescentus*. The *P. fluorescens* F113 contains an N-terminal YcgR domain (PF07317) and a C-Terminal PilZ domain (PF07238). This domain architecture is very similar to that of the YcgR protein. The DgrA protein also contains a PilZ domain. E-value indicates significance of the similarity to the PFAM model domain.

**Figure 2:**
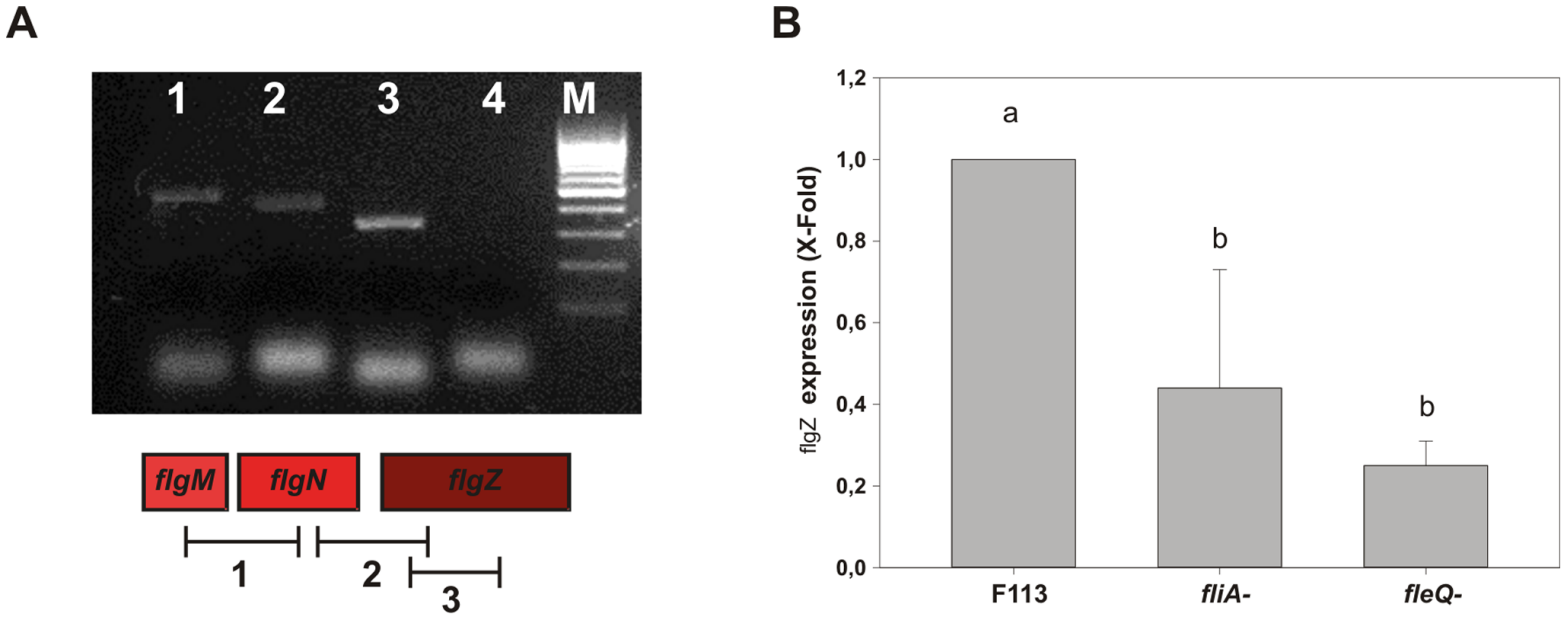
*flgZ* is a flagellar gene. A. *flgZ* is co-transcribed with the *flgMN* genes. RT-PCR analysis of cDNA from total RNA, obtained from *P. fluorescens* cultures in LB medium at O.D._600_ 0.8. Primers were designed to amplify the indicated fragments. Lanes 1 to 3 correspond to the numbered fragments. Lane 4 is a negative control which contains RNA that was not subjected to retrotranscription. The same expression pattern was found in cells growing in SA medium. B. *flgZ* expression is regulated by the flagellar regulatory genes *fliA* and *fleQ*. qPCR analysis of cDNA from total RNA, obtained from cultures of the wild type F113 strain and mutants affected in the *fliA* and *fleQ* genes in LB medium at O.D._600_ 0.8. Primers used amplified an internal region of the *flgZ* gene. Expression relative to F113 is shown as average plus standard deviation. Different letters indicates statistically significant differences (p<0.05).

The F113 chromosome harbors five genes that encode proteins with PilZ domains. One of these genes, termed here *flgZ* (PSF113_4460), is located downstream of the *flgMN* genes and in the same sense of transcription in the chromosome. This gene is highly conserved in sequence and synteny in the genomes of all sequenced pseudomonads (Fig. 1A). The *flgZ* gene encodes a protein belonging to the orthologous group COG5581 which contains the highly conserved PilZ domain in the C-terminus and a weakly conserved YcgR domain in the N-terminus (Fig. 1B), showing weak homology with the YcgR protein of Enterobacteria, a PilZ domain protein that has been shown to downregulate motility acting on the flagellar motor in response to the levels of c-di-GMP produced by several diguanylate cyclases [22], [23], [24]. The PilZ domain of *flgZ* shows also homology with a similar domain in the DgrA protein of *Caulobacter* [25] that has been also shown to downregulate swimming motility (Fig. 1B). The location of the *flgZ* gene adjacent to a cluster of previously identified flagellar genes, prompted us to test whether it was co-transcribed with these genes. Fig. 2A shows that *flgZ* is transcribed in a polycistronic mRNA together with *flgM* and *flgN*. Since it has been previously shown that in *P. aeruginosa flgMN* form part of the flagellar regulon and its transcription is weakly activated by FliA and FleQ [26], we tested the expression of *flgZ* in *fliA* and *fleQ* mutant backgrounds. As shown in Fig. 2B, mutations in *fliA* and *fleQ* reduced the expression level of *flgZ*. All these results show that *flgZ* is a flagellar gene whose expression is regulated within the flagellar regulon.

**Figure 3:**
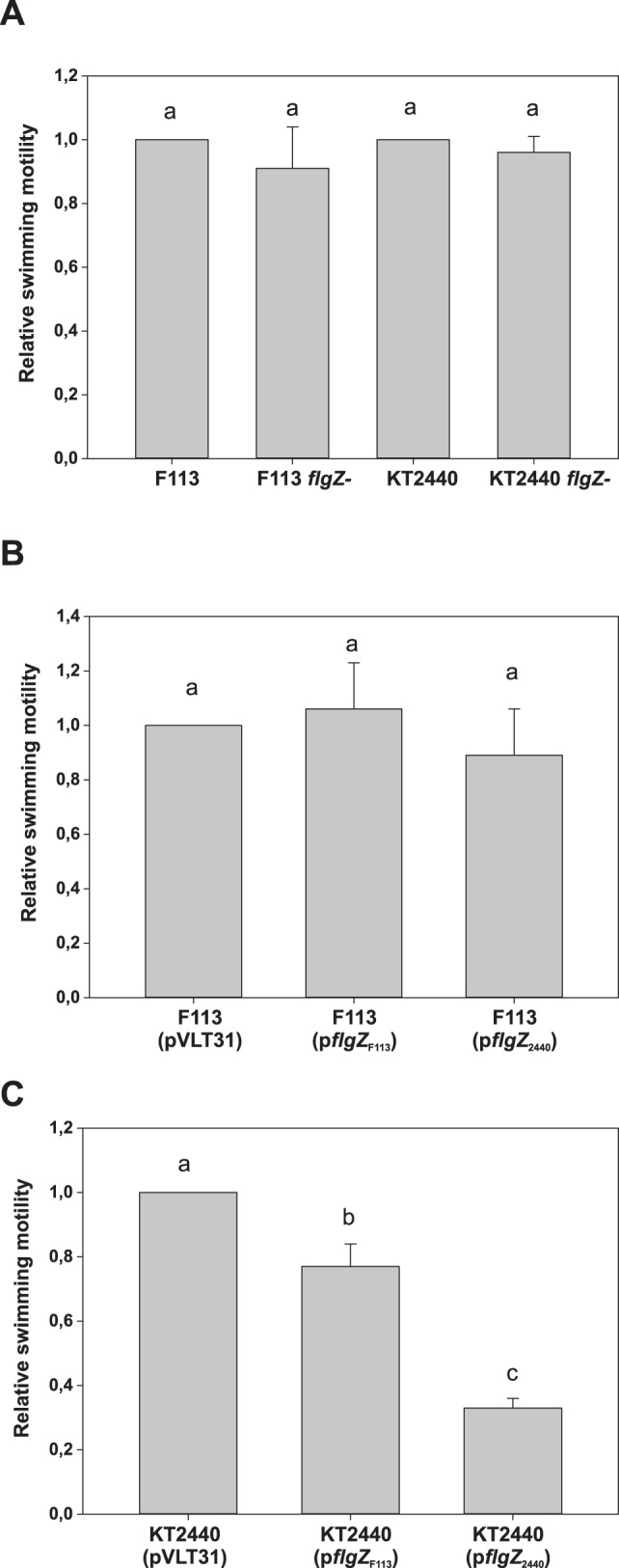
*flgZ* is implicated in the regulation of swimming motility. A. Relative swimming motility of *flgZ* mutants in *P. fluorescens* F113 and *P. putida* KT2440 backgrounds. B. Relative swimming motility of *P. fluorescens* F113 derivatives harboring plasmids which overexpress the F113 (p*flgZ*_F113_) and KT2440 (p*flgZ*_2440_) *flgZ* genes. C. Relative swimming motility of *P. putida* KT2440 derivatives harboring plasmids which overexpress the F113 (p*flgZ*_F113_) and KT2440 (p*flgZ*_2440_) *flgZ* genes. In B and C wild-type strains harbored the empty vector pVLT31 and *flgZ* expression was induced with 1mM IPTG. Swimming motility was determined by haloes diameter in SA plates with 0.3% agar, 24 hours after inoculation. Averages of three triplicated experiments plus standard deviation are shown. Different letters indicate statistically significant differences (p<0.05). Relative motility of each strain is also shown in Table S3.

In order to test whether FlgZ was implicated in the regulation of swimming motility, *flgZ* mutants were constructed by gene disruption in the *P. fluorescens* F113 and *P. putida* KT2440 backgrounds. Fig. 3A shows that the mutants did not present a swimming motility phenotype. We also tested the effect of overexpressing the *flgZ* gene. The F113 and KT2440 *flgZ* genes were independently cloned under a *Ptac* promoter in plasmid pVLT31. The resulting constructs, p*flgZ*_F113_ (pBG1837) and p*flgZ*_2440_ (pBG2004) were introduced in F113 and swimming motility was tested. As shown in Fig. 3B, *flgZ* overpression did not have an effect in swimming in strain F113. However, when the same constructs were introduced in KT2440, a significant reduction in swimming motility (Fig. 3C) was observed, indicating that in *P. putida*, *flgZ* participates in swimming motility regulation by reducing swimming activity. These results also show that the F113 *flgZ* gene is functional and that differences in activity depended on the genetic background and not in the *flgZ* gene.

### The Subcellular Localization of FlgZ Depends on c-di-GMP Levels

**Figure 4:**
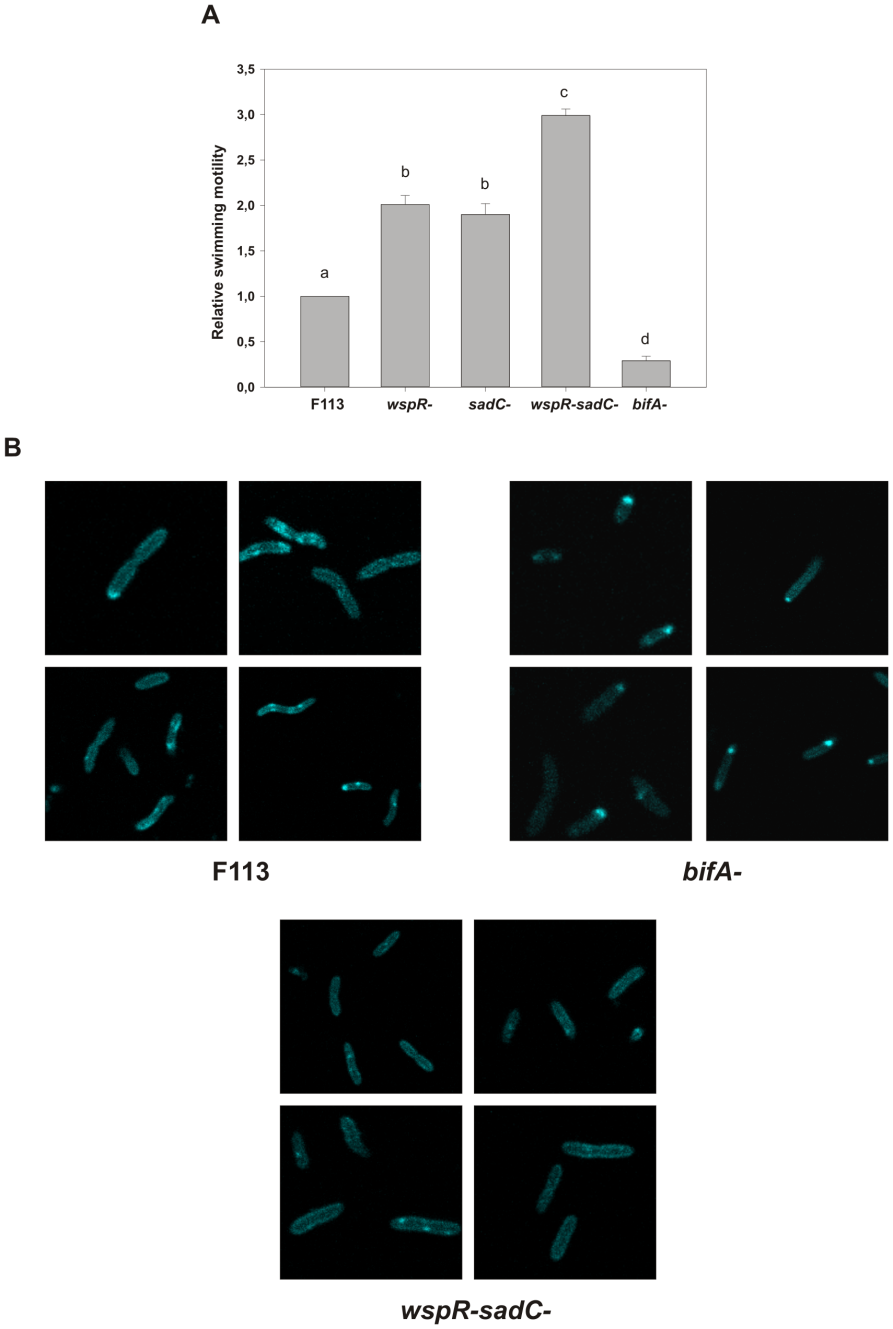
FlgZ subcellular localization is dependent on c-diGMP levels. A. Relative swimming motility of *P. fluorescens* F113 mutants affected in genes encoding diguanylate cyclases (*wspR* and *sadC*) and a phosphodiesterase (*bifA*). Swimming motility was determined by haloes diameter in SA plates with 0.3% agar, 24 hours after inoculation. Averages of three triplicated experiments plus standard deviation are shown. Different letters indicate statistically significant differences (p<0.05). Relative motility of each strain is also shown in Table S3. B. Subcellular localization of a fluorescent FlgZ-eCFP fusion protein in the wild-type (F113) strain and in derivatives with high (*bifA^-^*) and low (*wspR^-^sadC^-^*) c-di-GMP levels. The fusion protein is localized at the cellular pole in the *bifA* mutant, but patch distributed in the *wspRsadC* double mutant. An intermediate situation is observed in the wild-type strain. Representative confocal micrographies of exponentially growing cells in LB medium are shown.

PilZ domains have been demonstrated to participate in c-di-GMP sensing [4], [24]. Therefore we decided to test genes encoding diguanylate cyclases that had previously been shown to downregulate motility, for participation in *flgZ* signalling. The *wspR* gene encodes a diguanylate cyclase that is the output of the chemotaxis-like Wsp signal transduction pathway [27]. We have previously shown that in *P. fluorescens wspR* participates in flagellar motility downregulation, since a *wspR* mutant is hypermotile [16]. The SadC protein is a membrane protein which contains a GGDEF domain and has been shown to possess diguanylate cyclase activity and to affect motility in *P. aeruginosa* [6]. As shown in Fig. 4A, mutations in either of these genes in strain F113 resulted in increased swimming motility, confirming the role of both genes in downregulation of swimming motility. Furthermore, a double mutant *wspRsadC* showed an additive swimming phenotype, presenting higher motility than the single mutants. The BifA protein is a phosphodiesterase that has been shown to degrade c-di- GMP in *P. aeruginosa* [5]. As shown in Fig. 4A, mutation of *bifA* in *P. fluorescens* F113 resulted in reduced motility. These results clearly indicate a relation between c-di-GMP levels and swimming motility: the higher the amount of c-di-GMP the lowest the swimming motility ability of F113. Considering three possible levels of c-di-GMP levels: wt (physiological level), *bifA* mutant (increased c-di-GMP concentration) and *wspRsadC* mutant (reduced c-di-GMP concentration), we decided to test the subcellular localization of a FlgZ::eCFP fusion protein. As shown in Fig. 4B, the concentration of c-di-GMP dramatically affects the subcellular localization of the FlgZ fusion protein. Under the high c-di-GMP concentration present in the *bifA* mutant, the protein is localized to one of the cellular poles, the pole where flagella are located. The localization in the flagellar pole is especially evident in dividing cells. Conversely, under low c-di-GMP conditions, the protein appears in patches distributed through the whole cell. An intermediate situation is observed under c-di-GMP physiological conditions. These results are consistent with an interaction of the FlgZ protein with the flagellar basal body mediated by the concentration of c-di-GMP as it happens with the molecular brake YcgR in *E.coli* [23].

### c-di-GMP Levels Modulate Swimming Motility Independently of FlgZ

**Figure 5:**
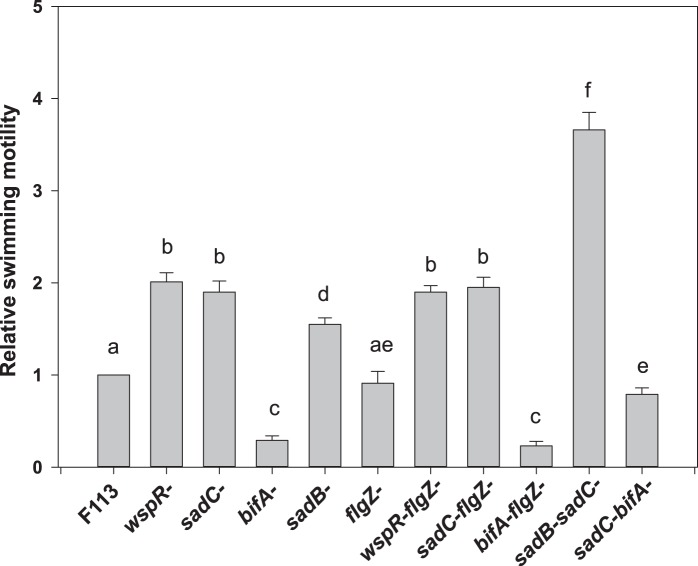
c-di-GMP levels modulate swimming motility independently of FlgZ. Relative swimming motility of *P. fluorescens* F113 mutants affected in genes encoding diguanylate cyclases (*wspR*, *sadC*), phosphodiesterase (*bifA*) and putative c-di-GMP sensing and signal transduction proteins (*flgZ* and *sadB*). The *flgZ* mutation did not suppress the swimming phenotypes of *wspR*, *sadC* and *bifA* mutants. Swimming motility was determined by haloes diameter in SA plates with 0.3% agar, 24 hours after inoculation. Averages of three triplicated experiments plus standard deviation are shown. Different letters indicate statistically significant differences (p<0.05). Relative motility of each strain is also shown in Table S3.

In order to test the implication of *flgZ* in the regulation of swimming through c-di-GMP, we constructed double mutants affected in *flgZ* and either of the tested genes encoding enzymes implicated in c-di-GMP turnover. Fig. 5 shows that both the *wspRflgZ* and the *sadCflgZ* double mutants showed a swimming phenotype that was not significantly different from the swimming phenotype of the *wspR* and *sadC* mutants. Furthermore, a *bifAflgZ* double mutant also showed a swimming phenotype identical to the phenotype of the *bifA* mutant. These results show that signal transduction leading to swimming motility downregulation, mediated by c-di-GMP generated by WspR and SadC and degraded by BifA is, at least, partially independent of *flgZ*. Since in *P. aeruginosa* it has been suggested that the c-di-GMP produced by SadC might be sensed through SadB, we also constructed double mutants affected in *sadB* and *sadC*, and in *sadC* and *bifA*. As shown in Figure 4, the *sadCbifA* mutant had an intermediate swimming phenotype reflecting the antagonic effect of both genes. However, the *sadBsadC* mutant showed an additive phenotype presenting higher motility than the individual mutants. We have previously shown that a *sadBwspR* mutant also have an additive swimming phenotype [16]. These results show that in *P. fluorescens* F113, neither SadC nor WspR act through SadB in regulating swimming motility.

### c-di-GMP Modulates Biofilm Formation through FlgZ

**Figure 6:**
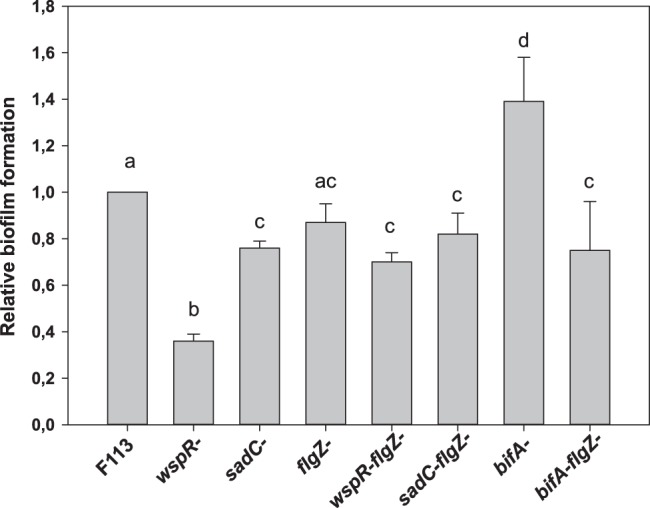
c-di-GMP modulates biofilm formation through FlgZ. Relative biofilm formation by *P. fluorescens* F113 mutants affected in c-di-GMP turn-over and sensing. The *flgZ* mutation suppressed the biofilm forming phenotype of mutants affected in genes encoding the WspR diguanylate cyclase and the BifA phosphodiesterase. Biofilm formation was determined colorimetrically from the crystal violet staining biomass attached to plastic in 96 wells plates, after elimination of the culture grown in these wells in LB medium for 8 h. Averages of three experiments with eight replicates plus standard deviation are shown. Different letters indicate statistically significant differences (p<0.05).

Since regulation of flagellar motility in pseudomonads by c-d-iGMP seems to be opposed to regulation of biofilm formation [28] and the *wspR* mutant in *P. fluorescens* is defective in biofilm formation [16], we decided to test whether *flgZ* participates in this process. As shown in Fig. 6, while the *sadC* and *wspR* mutants form less biofilm (as biomass attached to plastic) than the wild-type strain, the *flgZ* mutant does not significantly differ from the wild-type strain for biofilm formation. However, double mutants *flgZsadC* and *flgZwspR* do not differ significantly from the *flgZ* mutant. Furthermore, a mutation affecting *flgZ* is suppressing the biofilm forming phenotype of the *wspR* mutant. On the other hand, the *bifA* mutant forms significantly more biofilm than the wild-type strain and the *flgZ* mutant and this enhanced biofilm formation phenotype is suppressed in the *bifAflgZ* double mutant, where *flgZ* values are restored. These results show epistasis of *flgZ* over *wspR* and *bifA*, indicating that the effect of this diguanylate cyclase and phosphodiesterase in biofilm formation is mediated through FlgZ. Therefore, FlgZ is a new c-di-GMP binding protein implicated in biofilm formation.

## Discussion

Opposed sessile and planktonic lifestyles in bacteria have been associated with the intracellular levels of c-di-GMP [28]. The current paradigm indicates that high levels of this messenger are associated with a sessile lifestyle characterized by the formation of biofilms, while low c-di-GMP levels promote biofilm dispersal and swimming motility [29], [30], [31]. Perception of c-di-GMP has been associated with several protein domains [32], [33] and with specific structures in mRNA (riboswitches) [34], [35]. One of the protein domains that has been demonstrated to bind c-di-GMP across the bacterial kingdom is the PilZ domain [4], [24], originally described in cellulose synthases [36], [37]. The pseudomonads contain several proteins with PilZ domains. The gene encoding one of these proteins, here named *flgZ*, is present in all the pseudomonadś genomes sequenced so far and the degree of sequence conservation and genomic context indicate that these genes are true orthologs. The *Pseudomonas putida* KT440 FlgZ protein has been purified and its crystal structure has been determined to show that FlgZ binds two molecules of c-di-GMP [38]. Binding of c-di-GMP induces dimer to monomer transition, suggesting that the monomeric state is the active form [38].

Several of the results presented here indicate that FlgZ is implicated in motility regulation. FlgZ shows homology with the enterobacterial YcgR protein that has been shown to modulate motility, acting as a flagellar brake in response to c-di-GMP [24], [39]. FlgZ and YcgR share the same domain architecture, with a C-terminal PilZ domain and an N-terminal YcgR domain. Furthermore, we have shown here that *flgZ* is located in a flagellar operon and is co-transcribed with the flagellar *flgMN* genes. Transcription of *flgZ* depends on the flagellar regulators FleQ and FliA. Similarly to Enterobacteria, in *P. fluorescens* F113 mutation of the *flgZ* gene does not alter the swimming motility phenotype [24] but ectopic expression of two *flgZ* orthologs reduce swimming motility in *P. putida*. Finally, the location of FlgZ in the flagellar pole of the cell under high c-di-GMP conditions may indicate interaction with the flagellar basal body as it has been shown for YcgR. Taken together, these results indicate that FlgZ may modulate swimming motility in pseudomonads by c-di-GMP dependent interaction with the flagellar rotor, similarly to the function of YcgR in Enterobacteria.

The genome of *P. fluorescens* F113 encodes more than thirty proteins with domains involved in c-di-GMP turn-over [20], [21]. We have previously shown that the WspR protein, which has a diguanylate cyclase activity, is regulating swimming motility through a pathway that does not affect flagella biosynthesis [16]. WspR also affects biofilm formation, since a *wspR* mutant produces less biofilm than the wild-type strain [12]. Here we have tested mutants in other two genes, encoding another diguanylate cyclase, *sadC* [6] and a c-di-GMP specific phosphodiesterase, *bifA* [5]. The *sadC* mutant showed increased swimming motility, presenting a motility phenotype very similar to the *wspR* mutant phenotype. This result indicates that in F113, SadC is implicated in swimming motility regulation. Furthermore, a double mutant *sadCwspR* presents an additive phenotype, since this double mutant shows higher swimming motility than either of the individual mutants. This suggests that c-di-GMP synthesized by both enzymes contributes independently to motility regulation. The *bifA* mutant presented a severe decrease in swimming motility, indicating that swimming motility is activated by BifA probably by depleting intracellular levels of c-di-GMP, in agreement with the c-di-GMP paradigm. We have also shown this antagonist role, since the *bifAsadC* mutant presented an intermediate swimming phenotype. However, when we tested the swimming motility phenotype of double mutants simultaneously affected in *flgZ* and in any of the other genes, *wspR*, *sadC* or *bifA*, we found that the *flgZ* mutation did not alter the phenotype of the individual mutants. This result indicates that there are additional factors besides FlgZ that influence c-di-GMP regulation of swimming motility. A similar result has been found in *Salmonella*, where this additional factor was identified as cellulose production [40]. However, *P. fluorescens* F113 should not produce cellulose, since its genome lacks the cellulose synthase operon *wss* [20] that is present in other *P. fluorescens* strains such as SBW25 [19]. It has been suggested that modulation of motility and biofilm formation by SadC in *P. aeruginosa* is partly mediated by the production of the Pel exopolysaccharide [41]. Since the *pel* operon is also absent from the F113 genome [20], it is possible that a yet unidentified polysaccharide is partly responsible of the swimming behaviour of strain F113.

The availability of the *bifA* and the *sadCwspR* mutants, allowed us to test the subcellular localization of the FlgZ protein under different c-di-GMP intracellular concentrations. When we observed the localization of an FlgZ protein fused to a fluorescent protein, we found that the localization indeed depended of c-di-GMP levels. Under high c-di-GMP levels (*bifA* mutant), most of FlgZ was located at a spot at the flagellar pole of the cell, suggesting that under these conditions FlgZ may interact with the flagellar basal body. Conversely, under low c-di-GMP conditions (*sadCwspR* mutant) the FlgZ protein is patchly distributed through the bacterial cytoplasm and therefore not interacting with the flagellar structure. Since c-di-GMP binding by FlgZ induces a dimer to monomer transition [38], it is likely that monomerization is required for FlgZ localization and functioning. It has been previously shown in *P. aeruginosa* that activation by phosphorylation of WspR induces subcellular clustering of the protein [42]. All these findings highlight the importance of subcellular compartmentalization for c-di-GMP function.

As indicated above, WspR is implicated in biofilm formation. We have shown here that the *sadC* and *sadCwspR* mutants are also impaired in biofilm formation. The biofilm formation defect is more severe in the double mutant, suggesting again that c-di-GMP produced by both diguanylate cyclases independently contribute to biofilm building. Conversely, the *bifA* mutant produced a biofilm significantly denser than the wild-type strain. These results show that in *P. fluorescens* F113 these three enzymes participate in c-di-GMP mediated regulation of biofilm formation. Biofilm formation was not affected in a *flgZ* mutant. However, the *flgZ* mutation was shown to suppress the biofilm forming phenotype of the *sadC*, *wspR* and *bifA* mutations, since all the double mutants showed a biofilm forming phenotype not different from the *flgZ* mutant and wild-type phenotypes. The epistatic relation of *flgZ* over the other genes indicates that the c-di-GMP produced by WspR and SadC and degraded by BifA under physiological conditions modulates biofilm formation through FlgZ. A role for c-di-GMP for biofilm formation in pseudomonads has been previously shown for the stability of the adhesin LapA, mediated by the c-di-GMP sensed by the non-PilZ protein LapD [9]. Here we describe a novel activity exerted by the c-di-GMP sensed by FlgZ which also modulates biofilm formation in pseudomonads.

## Methods

### Strains, Plasmids and Growth Conditions

Strains and plasmids used are listed in Table S1. Strains of *Pseudomonas* were grown on SA medium [43] at 28°C, 1.5% purified agar (Pronadisa) was used for solid medium. When necessary, antibiotics were used at the following concentrations (µg/mL): Rifampicin (Rif) 100; Kanamycin (Km) 50; Gentamycin (Gm) 5; Spectomycin (Spc) 100. *E. coli* strains were grown on LB medium at 37°C [44]. Antibiotics were used at the following concentrations (µg/mL): Kanamycin (Km) 25; Gentamycin (Gm) 10; Spectomycin (Spc) 100; Ampicillin (Amp) 100.

### DNA and RNA Manipulations

Routine DNA manipulations were performed using standard procedures. Plasmid DNA from *E. coli* was purified using the Wizard® Plus SV Minipreps DNA purification system plasmid kit (Promega). Plasmids were transformed into *E. coli* by heat-shock transformation. Restriction enzymes were purchased from Takara and used according to the manufacturer’s instructions. Oligonucleotides are listed in Table S2 of the supplemental material. PCR fragments were cloned pGEM -T Easy vector (Promega). Mutants were obtained either by Tn5 mutagenesis using pCAM140 [45] or by single homologous recombination of amplified internal fragment from the gene cloned in pK19*mobsacB* [46] or pGmob2 [47] suicide vectors. Mutants were checked by Southern blotting and by PCR. Overexpression of *flgZ* genes was achieved by cloning them under the control of the IPTG-inducible promoter present in the pVLT31 plasmid [48]. The translational fusion FlgZ-eCFP was generated by adding to the *flgZ* 3′end and to the cyan-*ecfp* 5′end a PstI restriction site using primers described in Table S1, in order to ligate both genes and the resulting fragment was cloned in the expression vector pVLT31 [48]. Plasmids were mobilized into *P. fluorescens* F113 [49] or *P. putida* KT2440 [50] by triparental mating, using pRK2013 as the helper plasmid [51].

Total RNA was extracted using Trizol according to manufacturer’s specifications (Invitrogen) from *P. fluorescens* strains grown to 0.8 O.D._600_ in LB medium. Genomic DNA remains were removed by RQ1 RNase-Free DNase treatment (Promega) for 30 minutes at 37°C. After that, RNA was purified using Trizol. The concentration of RNA was spectrophotometrically determined in a Nanodrop and integrity was verified in denaturing agarose gels. All RNA samples were stored at −80°C. cDNA was synthetized from 1 µg of extracted RNA using SuperScriptIII First-Strand Synthesis System following manufacturer’s instructions (Invitrogen). RT-PCR analysis was done using 1 µl of cDNA synthesized and primers listed in Table S2.

### Confocal Laser Scanning Microscopy

Images were captured in a Leica TC5 SP5 confocal microscope equipped with 63× objective and eCFP was excited with a 458 nm Laser from argon line. The emission was detected using a 475 nm band pass filter.

### Swimming Assays

SA medium plates containing 0.3% purified agar were used to test swimming motility. Cells from exponentially growing cultures were inoculated into the plates using a toothpick. Swimming haloes were measured after 24 h of inoculation. Every assay was performed three times with three replicates each time.

### Biofilm Assays

Biolfim assays were performed using the same procedure as that described by Fletcher [52] with some modifications described by Barahona *et al*. [12]. Briefly, 100 µL from exponentially LB medium-growing cultures adjusted to O.D._600_ = 0.04 were placed on 96-well microtiter plates and incubated for 8 h at 28°C. 25 µL of a 1% (w/v) crystal violet (CV) was added to each well for 15 min at room temperature for staining of adhered cells. Dye excess was eliminated by rinsing with distillated water. 200 µL of 95% ethanol was added to each well in order to extract CV from cells, extraction was performed overnight at room temperature with shaking. Absorbance was measured in a microtiter plate reader at 590 nm. Every assay was performed three times with eight replicates each time.

### Bioinformatic Analysis

Whole *P. fluorescens* F113 proteome was checked against PFAM database to find out proteins containing PilZ domains. Results were compared to other described PilZ proteins to correlate domain organization. Other *Pseudomonas* orthologs to PSF113_4460 (FlgZ) were searched with BLASTn [53], keeping hits with a match percentage cut-off 50% and an expected value of 1e-5. Genome data from results appearing in fully sequenced *Pseudomonas* were obtained from NCBI ftp server. 20 Kb nucleotide annotation data around orthologs were checked for synteny studies. ORFs with no annotation were assigned by BLASTx against NCBI nr database and RAST prediction [54].

### Statistical Methods

SigmaStat software package (Systat software) was used for all statistical analyses. The data were compared using one way analysis of variance (ANOVA) followed by Bonferroni’s multiple comparison test (p<0.05).

## Supporting Information

Table S1 Strains and Plasmids.
(DOC)

Table S2 Primers used.
(DOC)

Table S3 Relative motility of strains related to F113 and KT2440 wild-type strains.
(DOC)
