# Supplementary material for: Identification of flgZ as a Flagellar Gene Encoding a PilZ Domain Protein That Regulates Swimming Motility and Biofilm Formation in Pseudomonas
Source: PLoS One. 2014 Feb 4;9(2):e87608. doi: 10.1371/journal.pone.0087608 (PMC3913639; doi:10.1371/journal.pone.0087608)
Supplement: Table S1 — Strains and Plasmids. (DOC) [file pone.0087608.s001.doc]

| Strains and Plasmids | Characteristics | Source or Reference |
| --- | --- | --- |
| **STRAINS**  ***- P. fluorescens*** |  |  |
| F113 Rif | *P. fluorescens* wt Rif^R^ | [49] |
| F113*fleQ* | F113 Rif fleQ^-^ Rif^R^Km^R^ | [13] |
| F113*fliA* | F113 Rif *fliA*^-^ Rif^R^Km^R^ | This work |
| F113*sadB* | F113 Rif *sadB^-^* Rif^R^Km^R^ | [19] |
| F113*sadC* | F113 Rif *sadC^-^* Rif^R^Gm^R^ | This work |
| F113*bifA* | F113 Rif *bifA^-^* Rif^R^Gm^R^ | This work |
| F113*flgZ* | F113 Rif *flgZ^-^* Rif^R^Gm^R^ | This work |
| F113*wspR* | F113 Rif *wspR^-^* Rif^R^Spc^R^ | This work |
| F113*wspR-flgZ* | F113 Rif *wspR^-^flgZ^-^* Rif^R^Gm^R^Spc^R^ | This work |
| F113*sadC-flgZ* | F113 Rif *sadC^-^flgZ^-^*  Rif^R^Gm^R^Km^R^ | This work |
| F113*bifA-flgZ* | F113 Rif *bifA^-^flgZ^-^*  Rif^R^Gm^R^Km^R^ | This work |
| F113*sadC-sadB* | F113 Rif *sadC^-^sadB^-^*  Rif^R^Gm^R^Km^R^ | This work |
| F113*sadC-bifA* | F113 Rif *sadC^-^bifA^-^* | This work |
|  | Rif^R^Gm^R^Km^R^ |  |
| F113*sadC-wspR* | F113 Rif *sadC^-^wspR^-^* | This work |
| ***- P. putida*** |  |  |
| KT2440 | *P. putida wt* | [50] |
| KT2440*flgZ* | KT2440*flgZ^-^* Km^R^ | This work |
| **PLASMIDS** |  |  |
| pGEM-T Easy vector | Cloning vector Amp^R^ | Promega |
| pRK2013 | Helper plasmid Km^R^ | [51] |
| pK19*mobsacB* | Suicide SacB Km^R^ | [46] |
| pG18*mob*2 | Suicide vector Gm^R^ | [47] |
| pCAM140 | Vector with miniTn5*gus*Spc^R^ | [45] |
| pVLT31 | Expression vector with *Ptac*  inducible promoter | [48] |
| pBG2004 | pVLT31 with *flgZ* from KT2440 | This work |
| pBG1837 | pVLT31 with *flgZ* from F113 | This work |
| pBG1923 | pVLT31 with *flgZ-eCFP* | This work |
|  |  |  |
