# Supplementary material for: Identification of flgZ as a Flagellar Gene Encoding a PilZ Domain Protein That Regulates Swimming Motility and Biofilm Formation in Pseudomonas
Source: PLoS One. 2014 Feb 4;9(2):e87608. doi: 10.1371/journal.pone.0087608 (PMC3913639; doi:10.1371/journal.pone.0087608)
Supplement: Table S2 — Primers used. (DOC) [file pone.0087608.s002.doc]

| Primer | Sequence5’→ 3’a | Use |
| --- | --- | --- |
| FliAF2  FliAR | CTGGTCAAACGCATCGCCT  CGCACTGGCTGTGCAACC | - fliA mutant construction |
| BifAF  BifAR | GCTTGTCTGTGAAGTTGCTC  AAGCCATGCGTACGGCTTC | - bifA mutant construction |
| FlgZF113F  FlgZF113R | CCAGAGCTATCTGGTCAACG  GAAATGTCCAGCAGCTTGCC | - F113 flgZ mutant construct. |
| FlgZKT2440F  FlgZKT2440R | GAAAGCAACACCCTGGCACTGG  CCCGCCTTGAAGCGCTCATAC | - KT2440 flgZ mutant construct. |
| SadBF1  SadBR1 | ATCATCAAGCTGCGGGTCG  CAGGTGCTGTTCCACGTAG | - sadB mutant construction |
| SadCF  SadCR | TCATTACCTCGACTCGTTGC  ATGCGCTTGAAATGGTCGAG | - sadC mutant construction |
| WspRF1  WspRR3 | CTGATGGCCGAGAAACTGC  ACATCCAGGGTGATTTATTCG | - wspR mutant construction |
| FlgZextF113F2  FlgZextF113R2 | GTGTTCAATGCCTCAAACGC  TCAGAGGTCGTCTTTATCGAA | - F113 *flgZ* amplification |
| FlgZextKT2440F  FlgZextKT2440R | TGTGTCGCTTTTGCCTGGAG  AAGCTTCCGCGGGTGGATGAGCA | - KT2440 *flgZ* amplification |
| PstI-eCFPF  eCFPR | ATCCTGCAGCTGAGCAAGGGCGAGGAGCTG  TTACTTGTACAGCTCGTCCATGCC | *- eCFP*  amplification with PstI site |
| FlgZextF113F2  FlgZR2-PstI | GTGTTCAATGCCTCAAACGC  ATCCTGCAGGAGGTCGTCTTTATCGAA | - F113*flgZ* amplification with PstI site |
| qFlgMF  pFlgNR | AACAAGTCAGCAACGGGGAGT  GTTGAGGCTGGCGAGGATCT | *- flgMN* amplification |
| qFlgNf  pFlgZR | GCCAGTCGATCCTGATGCAA  CAGGGCAATGGATTGGTCTC | *- flgNZ* amplification |

^a^PstI restriction site is underlined in primer sequence
