# Supplementary material for: Identification of flgZ as a Flagellar Gene Encoding a PilZ Domain Protein That Regulates Swimming Motility and Biofilm Formation in Pseudomonas
Source: PLoS One. 2014 Feb 4;9(2):e87608. doi: 10.1371/journal.pone.0087608 (PMC3913639; doi:10.1371/journal.pone.0087608)
Supplement: Table S3 — Relative motility of strains related to F113 and KT2440 wild-type strains. (DOC) [file pone.0087608.s003.doc]

| Strains | Motility (Mean±SD) |
| --- | --- |
| ***- P. fluorescens*** |  |
| F113 wild-type | 1 |
| F113 *flgZ-* | 0.91±0.13 |
| F113 p*flgZ*_F113_ | 1.06±0.17 |
| F113 p*flgZ*_2440_ | 0.89±0.17 |
| F113 *wspR-* | 2.01±0.1 |
| F113 *sadC-* | 1.90±0.12 |
| F113 *wspR*-*sadC-* | 2.99±0.07 |
| F113 *bifA-* | 0.29±0.05 |
| F113 *sadB-* | 1.55±0.07 |
| F113 *wspR-flgZ-* | 1.90±0.07 |
| F113 *sadC-flgZ-* | 1.95±0.11 |
| F113 *bifA-flgZ-* | 0.23±0.05 |
| F113 *sadB-sadC-* | 3.66±0.19 |
| F113 *sadC-bifA-* | 0.79±0.07 |
| ***- P. putida*** |  |
| KT2440 | 1 |
| KT2440 *flgZ-* | 0.96±0.05 |
| KT2440 p*flgZ*_F113_ | 0.77±0.07 |
| KT2440 p*flgZ*_2440_ | 0.33±0.03 |
